# Supplementary material for: Private capital holding, financial awareness of government and steadying operation of banks: Evidence from China
Source: PLoS One. 2023 May 11;18(5):e0285588. doi: 10.1371/journal.pone.0285588 (PMC10174543; doi:10.1371/journal.pone.0285588)
Supplement: Attachment — Submitted filename: Review opinions.pdf [file pone.0285588.s002.pdf]

审稿意见：大修

问题 1：面板数据中采用的是 123 家城市建设银行作为个体，那么 Finawa（政府的金融意识）的数据是如何收集和得到的？作者说通过银行、保险、证券、基金和信托 5 类金融机构的年报得到，那这 5 类金融机构和 123 家城市建设银行有关联吗，数据能一致吻合吗？麻烦作者给予解释。同理，作者是如何将 GDP 和城市级别的建设银行个体联系在一起？请作者回答。审稿人的意思是本文采用的是银行-城市级面板数据，还是银行级面板数据？

问题 2：此外，如果是银行-城市级面板数据，GDP 的数据应具体到城市级，而不是通过国家统计局获得省级面板数据，麻烦从《中国城市统计年鉴》重新找数据进行匹配（据审稿人了解，应该是可以找到这部分数据）。

建议 1：既然 Financial awareness of government（Finawa）是通过 Private capital holding（Priv）对 steady operation of banks（ln-Z）起作用，即 Finawa 发挥的是中介作用，所以在“2Literature review and research hypothesis”中的第 2 小节，不应该是“Financial awareness of government and steady operation of banks”，请改正第 2 小节的标题，修改研究假设 2，并扩充理论分析。

建议 2：本文采用 SYS-GMM 方法，主要解决被解释变量与滞后被解释变量的内生性，无法解决重要变量遗漏问题，加入控制变量可以解决遗漏变量导致的内生性问题，所以请作者改正这个说法。本文也加入了控制变量，请在那里说明可以解决遗漏变量导致的模型内生性问题。

建议 3：本文在“measures”中说加入了时间趋势项 YEAR，用于控制时间效应，但在“模型表达式”中没有（最好不要包含在控制变量中），麻烦加上，以便使模型表达式更完整。

建议 4：本文采用 SYS-GMM，所以对 SYS-GMM 的适用条件是否适用应该有所交代，麻烦作者给予补充（如：随机扰动项自相关性检验、过度识别检验）。

建议 5：Table A1.Variable definitions 应该出现在正文中，而不是附录中，这样有利于读者的理解。建议增加在“3 Data and methods”中，并且增加“变量说明”这一小节，在这一小节详细说明 1）被解释变量；2）核心解释变量；3）控制变量，并附加 Table A1.Variable definitions，以便读者对变量选取有详细的理解。（比如，被解释变量 ln_Z 是怎么得到的，虽然作者在“results”中有少量阐述 ，但麻烦在“变量说明”这一小节说明，不至于杂乱无章）

建议 6：麻烦在“4Results and discussion”中增加一小节“变量数据的平稳性检验”，使得实证结果分析更完整（虽然短面板数据基本平稳）。

建议 7：一般来讲，使用 SYS-GMM 后，内生性问题就基本解决了。但作者又提到了“核心解释变量与被解释变量的可能的互为因果的内生性问题”，既然考虑到了，烦请增加一部分“6 内生性的说明”，请在这一部分进行阐述。

建议 8：“5 Robustness checks”中采用替换核心解释变量的方法（找了 2 个替换变量）进行稳健性检验，麻烦作者分两小节解释，以便让读者更清楚明了。

建议 9：Table2—Table5 中的“Note”一般放在表格下面，请更正。

建议 10：“Conclusion and Enlightenment”中应增加一小节“未来展望”，即“未来展望”应包含在内。

建议 11：请注意修正文中翻译错误（如 1、“摘要”中最好用现在时态，而不是过去时态；2、**第 28 行**：We attempts to illustrate that the introduction of private capital is conducive to the release of the potential efficiency of the CCBs, but the result is closely linked with the financial awareness of the government.；3、**第 34 行:** we sets up the index of government financial awareness based on the data of the top ten shareholders of 123 CCBs from 2007 to 2017 in China. 不应该是第三人称单数；等等）和规范本文参考文献格式。

Review comments: ***major revision***

Question 1: 123 city construction banks are used as individuals in the panel data. Then how is the data of “Finawa” (the government's financial awareness) collected and obtained? The author said it was obtained from the annual reports of 5 financial institutions: banks, insurances, securities, funds and trusts. Are these 5 financial institutions related to 123 city China Construction banks? Please explain. Similarly, how does the author relate GDP to individual city construction banks? Please answer. Does the reviewer mean that this paper uses bank-city-level panel data or bank-level panel data?

Question 2: If it is bank-city level panel data, the data of GDP should be specific to city level instead of provincial level panel data obtained by National Bureau of Statistics. Please kindly find data from “China City Statistical Yearbook” for matching (according to the reviewer, this part of data should be found).

Suggestion 1: Since “Financial Awareness of Government” (Finawa) works on “the steady operation of banks” (ln-Z) through “Private capital holding” (Priv), That is, it (Finawa) plays an intermediary role, so in subsection 2 of "2Literature review and research hypothesis", It should not be "Financial awareness of government and steady operation of banks", please correct the title of subsubsection 2., modify Hypothesis 2 and modify the theoretical analysis.

Suggestion 2: SYS-GMM method was adopted in this paper to mainly solve the endogeneity of explained variables and lagged explained variables, which could not solve the problem of omission of important variables. Adding control variables could solve the endogeneity problem caused by missing variables. Therefore, please correct this statement. In addition, control variables are also included in this article. Please explain there that model endogeneity problems caused by missing variables can be solved.

Suggestion 3: In "measures", the author says that the time trend item YEAR is added to control the time effect, but it is not included in the "model expression" (YEAR is not included in the control variable). Please add it in order to make the model expression more complete.

Suggestion 4: SYS-GMM is used in this paper, so whether the applicable conditions of SYS-GMM are applicable should be explained. Please kindly give a supplementary explanation (such as: automatic correlation test of random disturbance term, over-recognition test).

Suggestion 5: Table A1.Variable definitions should appear in the text, not in the appendix. Only in this way can readers understand. It is suggested to add "3 Data and methods", and add a subsection of "Variable Description", in which details are explained: 1) explained variables;2) Core explanatory variable;3) Control variables, and attach “Table A1.Variable definitions”, so that readers have a detailed understanding of variable selection.(For example, how the explained variable ln_Z was obtained, although the author explains a little in the "results" section, please explain in the "variable description" section.)

Suggestion 6: Please add a subsection of "Stability test of variable data" to "4Results and discussion" to make the analysis of empirical results more complete (although the short panel data is basically stable).

Suggestion 7: In general, the endogeneity problem is basically solved using the SYS-GMM method. However, the author also mentioned "the endogeneity problem that the core explanatory variable and the explained variable may be causal to each other". Since this is taken into consideration, please kindly add a part of "6 Endogeneity explanation", and please elaborate in this part.

Suggestion 8: In "5 Robustness checks", the method of replacing core explanatory variables (two replacement variables are found) is used to test robustness. Please kindly ask the author to explain it in two subsections, so that readers can be clearer.

Suggestion 9: "Note" in Table2-Table5 is usually placed under the table, please correct them.

Suggestion 10: A subsection "Future prospects" should be added to "Conclusions and Enlightenments", that is, "Future prospects" should be included.

Suggestion 11: Please be careful to correct translation errors (for example, 1)it is better to use the present tense in the "abstract" rather than the past tense; 2) Line 28: We attempts to illustrate that the introduction of private capital is conducive to the release of the potential efficiency of the

CCBs, but the result is closely linked with the financial awareness of the government.; 3) Line 34: we sets up the index of government financial awareness based on the data of the top ten shareholders of 123 CCBs from 2007 to 2017 in China. etc.) and standardize the reference format of the article.
