# Supplementary material for: Private capital holding, financial awareness of government and steadying operation of banks: Evidence from China
Source: PLoS One. 2023 May 11;18(5):e0285588. doi: 10.1371/journal.pone.0285588 (PMC10174543; doi:10.1371/journal.pone.0285588)
Supplement: Attachment — Submitted filename: Response to reviewers.docx [file pone.0285588.s003.docx]

****Response to reviewer #1** comments**

Dear Reviewer #1,

We are very grateful for your appreciation of our paper, which makes us very encouraged.

****Response to reviewer #2** comments**

Dear Reviewer Haisong Dong,

We really appreciate you for your carefulness and conscientiousness. Your suggestions are really valuable and helpful for revising and improving our paper. According to your suggestions, we have made the following revisions on this manuscript:

Question 1: 123 city construction banks are used as individuals in the panel data. Then how is the data of “Finawa” (the government's financial awareness) collected and obtained? The author said it was obtained from the annual reports of 5 financial institutions: banks, insurances, securities, funds and trusts. Are these 5 financial institutions related to 123 city China Construction banks? Please explain. Similarly, how does the author relate GDP to individual city construction banks? Please answer. Does the reviewer mean that this paper uses bank-city-level panel data or bank-level panel data?

**Response 1：**

First, the financial awareness of local governments (*Finawa*) is a provincial indicator.

In China, major financial institutions include banks, insurance institutions, securities, funds and trusts. According to the annual reports of various financial institutions in various provinces, we collect the information of ownership structure of each institution to obtain the amount of shares held by the local government, and calculate the assets held by the local government. Of course, we also need to add up the total assets of all financial institutions in the province to calculate the average share of financial assets held by local governments, namely, *Finawa*. The larger the share, the stronger the financial awareness of local governments and the more financial assets they hold.

Of course, these five types of financial institutions include local city commercial banks, but the difference is that *Finawa* is a provincial indicator, while the financial data of city commercial banks are bank-level indicators.

Finally, we should note that our data structure is the panel data at the bank level. Although the *Finawa* is a provincial indicator, it eventually matches each bank.

Question 2: If it is bank-city level panel data, the data of GDP should be specific to city level instead of provincial level panel data obtained by National Bureau of Statistics. Please kindly find data from “China City Statistical Yearbook” for matching (according to the reviewer, this part of data should be found).

**Response 2：**

This is our negligence. In fact, in China, the operation of city commercial banks is not limited to local cities, but all important cities in the province where they are located. This is also the reason why we choose the GDP of the province where the city commercial banks are located instead of the city GDP. We have added an explanation in the Measures section.

Suggestion 1: Since “Financial Awareness of Government” (Finawa) works on “the steady operation of banks” (ln-Z) through “Private capital holding” (Priv), That is, it (Finawa) plays an intermediary role, so in subsection 2 of "2Literature review and research hypothesis", It should not be "Financial awareness of government and steady operation of banks", please correct the title of subsubsection 2., modify Hypothesis 2 and modify the theoretical analysis.

**Response 1：**

We need to note that the government's financial awareness (*Finawa*) does not affect the steady operation of banks (*Ln_Z*) through private capital holding (*Priv*), that is, it is not an intermediary role. What we are talking about is the moderating effect. Based on the analysis of the impact of *Priv* on *Ln_Z*, we further studied whether *Finawa* changed the impact of *Priv*.

Suggestion 2: SYS-GMM method was adopted in this paper to mainly solve the endogeneity of explained variables and lagged explained variables, which could not solve the problem of omission of important variables. Adding control variables could solve the endogeneity problem caused by missing variables. Therefore, please correct this statement. In addition, control variables are also included in this article. Please explain there that model endogeneity problems caused by missing variables can be solved.

**Response 2：**

We have corrected the relevant statement and explained the role of adding control variables according to your suggestion.

Suggestion 3: In "measures", the author says that the time trend item YEAR is added to control the time effect, but it is not included in the "model expression" (YEAR is not included in the control variable). Please add it in order to make the model expression more complete.

**Response 3：**

We have added the variable *YEAR* to the model.

Suggestion 4: SYS-GMM is used in this paper, so whether the applicable conditions of SYS-GMM are applicable should be explained. Please kindly give a supplementary explanation (such as: automatic correlation test of random disturbance term, over-recognition test).

**Response 4：**

In the original manuscript, we simply explained that the model was proved to be reasonable through relevant tests. However, we have not specified which tests have been passed. We have revised them according to your suggestion.

Suggestion 5: Table A1.Variable definitions should appear in the text, not in the appendix. Only in this way can readers understand. It is suggested to add "3 Data and methods", and add a subsection of "Variable Description", in which details are explained: 1) explained variables;2) Core explanatory variable;3) Control variables, and attach “Table A1.Variable definitions”, so that readers have a detailed understanding of variable selection.(For example, how the explained variable ln_Z was obtained, although the author explains a little in the "results" section, please explain in the "variable description" section.)

**Response 5：**

We have added the subsection "Variable description" in the "Data and methods". Of course, we also attached a table of variable definitions in this subsection.

Suggestion 6: Please add a subsection of "Stability test of variable data" to "4Results and discussion" to make the analysis of empirical results more complete (although the short panel data is basically stable).

**Response 6：**

We added the stationarity test of variable data.

Suggestion 7: In general, the endogeneity problem is basically solved using the SYS-GMM method. However, the author also mentioned "the endogeneity problem that the core explanatory variable and the explained variable may be causal to each other". Since this is taken into consideration, please kindly add a part of "6 Endogeneity explanation", and please elaborate in this part.

**Response 7：**

We arranged the subsection of "Instrumental variable" in the original manuscript into a new section "Endogeneity explanation".

Suggestion 8: In "5 Robustness checks", the method of replacing core explanatory variables (two replacement variables are found) is used to test robustness. Please kindly ask the author to explain it in two subsections, so that readers can be clearer.

**Response 8：**

According to two different variables, we split the subsection of "Replace independent variable" into two subsections.

Suggestion 9: "Note" in Table2-Table5 is usually placed under the table, please correct them.

**Response 9：**

Indeed, the settings of "Note" for tables in different publications are different. We refer to similar articles published in PLOS ONE again (Liu et al., 2022a;2022b), and confirm the correct location of "Note".

Liu J, Xu L, Zhang Q. The influence of the largest private shareholder on bank loans: Evidence from China. PLoS ONE. 2022; 17(10): e0276877. https://doi.org/10.1371/journal.pone.0276877

Liu J, Zhang Q, Xiao C. Large private shareholders, industrial policies and industrial loans of city commercial banks: Evidence from China. PLoS ONE. 2022; 17(12): e0278654. https://doi.org/10.1371/journal.pone.0278654

Suggestion 10: A subsection "Future prospects" should be added to "Conclusions and Enlightenments", that is, "Future prospects" should be included.

**Response 10：**

We added a section "Future prospects" in "Conclusions and Enlightenments".

Suggestion 11: Please be careful to correct translation errors (for example, 1)it is better to use the present tense in the "abstract" rather than the past tense; 2) Line 28: We attempts to illustrate that the introduction of private capital is conducive to the release of the potential efficiency of the CCBs, but the result is closely linked with the financial awareness of the government.; 3) Line 34: we sets up the index of government financial awareness based on the data of the top ten shareholders of 123 CCBs from 2007 to 2017 in China. etc.) and standardize the reference format of the article.

**Response 11：**

We reviewed the grammar standards and related formats.

****Response to reviewer #3** comments**

Dear Reviewer #3,

We appreciate your recognition of our article. Your suggestions are very helpful to improve the quality of our paper. According to your suggestions, we have made the following revisions on this manuscript:

**More information should be included in the “Data” section.**

1. How were the 123 CCBs chosen? Are they representative of the underly population of CCBs in China?

**Response 1：**

As of January 2023, there are a total of 125 city commercial banks in China (very few of them have come from the restructuring of our sample banks). The information disclosure of some banks is not perfect (the scale is also small), so we can't get their relevant information, and finally we got 123 banks. Therefore, our sample represents almost all city commercial banks in China.

2. Why chose 2007-2017 as the study period? My concern is that this time period involves a time period for the global crisis. I am not sure how that event affects your key findings (--In particular, since the dynamic GMM model uses lagged variables and their differences as instruments for the endogenous variable, some of these lagged variables might have been contaminated with the global crisis). In fact, this issue should be checked in the “robustness checks” section.

**Response 2：**

On the one hand, city commercial banks began to disclose annual reports generally and in a standard manner in 2007. On the other hand, since 2018, some new city commercial banks have been established by restructuring several city commercial banks. These lead to our sample period of 2007-2017. Of course, this period includes the global crisis. However, in the process of indicator construction (the moving average of the three periods), most of the samples during the crisis were eliminated. By selecting the sample after 2008, the regression results are shown in the following table. It can be found that the column 1 and column 4 are the same as the regression results in the manuscript, while column 2 and column 3 are almost the same.

|  | (1) | (2) | (3) | (4) |
| --- | --- | --- | --- | --- |
|  | *Ln_Z* | *ROA* | *CAR* | Volatility of *ROA* |
| *Priv* | 1.5521^***^ | 0.5642^***^ | -0.1783^***^ | -0.2240^***^ |
|  | (5.94) | (3.04) | (-7.48) | (-3.31) |
| *Finawa* | -0.0886 | -0.1441 | 0.0024 | -0.1548^***^ |
|  | (-0.39) | (-1.30) | (0.46) | (-3.71) |
| *Priv×Finawa* | -1.6996^***^ | -0.5536^**^ | 0.2228^***^ | 0.1844^*^ |
|  | (-4.06) | (-2.36) | (7.13) | (1.87) |
| *Ln_Z_t-1_* | 0.8021^***^ |  |  |  |
|  | (20.47) |  |  |  |
| *ROA_t-1_* |  | 0.4248^***^ |  |  |
|  |  | (18.17) |  |  |
| *CAR_t-1_* |  |  | 0.1677^***^ |  |
|  |  |  | (20.88) |  |
| *σ(roa)_t-1_* |  |  |  | 0.3996^***^ |
|  |  |  |  | (14.28) |
| control variables/year | Yes | Yes | Yes | Yes |
| *N* | 466 | 638 | 660 | 413 |

3. There should be some paragraphs in the “Data” section to talk about your variables. For example, how are “stability” and “government awareness” measured? For example, how may a read understand what 1.944, the mean of “Ln_Z,” means? And why should it be taken logarithm? Yes, there are some explanations provided in Table A1, but I think variable definition and construction deserve a more formal place in the text to better inform the reader.
4. Table A1 can be incorporated into Table 1.

5. The first paragraph on page 6 (on what is included in the Z-scores) should be put in the Data or Model section.

**Response 3, 4 and 5：**

We have added the subsection "Variable description" in the "Data and methods" to talk about these variables. Of course, we also attached a table of variable definitions (Table A1) in this subsection. In addition, we have moved the description of Z-scores (the first paragraph on page 6 of the original manuscript) to the "Variable description" subsection.

6. Since you have data on the time dimension, I think a graph depicting the time trends of the key variables would be very helpful for understanding your story.

**Response 6：**

We have added some graphs in the "Descriptive Statistics" subsection to show the changes of some key variables in the time dimension.

**Modeling/results:**

1. The key explanatory variable, *Priv_it* is treated as a dummy variable. I wonder if it can be constructed as a continuous variable, i.e., the “share” of private capital. That way, more variation can be exploited in the regression.

**Response 1：**

We did not choose the “share” because different banks have different degrees of equity dispersion. In extreme cases, the shareholding ratio of the largest shareholder may be close to 50%, but it may only be about 5%. Therefore, we pay more attention to the actual holding capacity of private shareholders, that is, whether they are the largest shareholder. Of course, in the "Robustness checks" section, we constructed the proportion of private shareholders to conduct a robustness test, so that more changes are used in the regression.

2. Some of the explanatory variables are measured by their current values; some others are measured by their lagged values. More explanations should be provided to clear out the confusion. In fact, more discussions on why you selected your covariates (e.g., from theory or from the literature) may be added to the “Model” section.

**Response 2：**

The addition of lag term is the original setting of system GMM estimation (Shen and Yao,2008; Che et al.,2013). This is to solve the inertia of the dependent variable, that is, the current operating conditions may depend on the past operating conditions. We have explained in more detail in the “Model specification” subsection.

Shen Y, Yao Y. Does grassroots democracy reduce income inequality in China? Journal of Public Economics. 2008;92(10-11): 2182-2198. DOI: 10.1016/j.jpubeco.2008.04.002.

Che Y, Lu Y, Tao Z, Wang P. The impact of income on democracy revisited. Journal of Comparative Economics. 2013; 41(1): 159-169. https://doi.org/10.1016/j.jce.2012.05.006.

3. Other than simply saying that “a panel system GMM” model is estimated, it would be nice to provide some discussion on how this model achieves identification (—and why it is chosen over conventional panel data models?).

**Response 3：**

In the “Model specification” subsection, we added an explanation of why the system GMM model is selected. In the “Results” section, we add the description of relevant identification tests.

4. In fact, it would also be informative to compare results based on conventional panel-data models, such as fixed effects and random effects models, with those of the dynamic GMM models to see if the dynamic GMM indeed has a significant advantage over the conventional models.

**Response 4：**

We added fixed effects regression in the “Results” section to compare with GMM estimation of dynamic panel system.

5. The panel seems to be missing a lot of observations for some variables, as shown in Table 1. This might introduce a sample selection issue to the model—for example, note that column 1 of Table 2 has only 466 obs which is only 2/3 of the sample size in column 2. I wonder how this issue was addressed.

**Response 5：**

This is caused by three reasons. First, our panel data is unbalanced. Some variables have more observations, while others have less. The second is that GMM estimation of dynamic panel system involves lag term, which will also cause the loss of some observations. The third is the construction of *Ln_Z*, which is calculated by the moving average of the three periods before and after *ROA* and *CAR* and the volatility of *ROA*, which also leads to the observations of *Ln_Z* being less than others.

6. In robustness checks, the authors use the “regional average” of the endogenous variable as an instrumental variable for it. This might be inappropriate. This strategy exploits “peer effects” for identification, but note that any bank is also a peer for other banks – so the influence goes both way, which creates a reverse causality issue in the first stage regression. A better strategy would be to use lagged values of the peer-average measure to create instrument variables.

**Response 6：**

We used the lag term of the peer-average to create the instrument variable for robustness checks.

The writing needs careful revisions. There are many typos, grammatical errors, and style issues in the text. For example,
(1) Abstract: what is ROA? As a general rule, acronyms should be spelled out at their first appearance.
(2) The directions of many of the quotation marks are wrong.
(3) Lines 105-109: Please check the sentence. It reads very confusingly.
(4) Line 160 (page 5/21): “…a dummy variables, …” should be “… a dummy variable, …”
(5) Line 181: “onstability” should be “on stability.”

**Response：**

We reviewed the spelling, grammar and style of the manuscript again and corrected the corresponding errors and deficiencies.

****Response to reviewer #4** comments**

Dear Reviewer #4,

We are very grateful for your favorable view on our manuscript. Your comments are very important to the acceptability of our paper. According to your suggestions, we have made the following revisions on this manuscript:

(1) The paper has made great contributions to the body of knowledge, but in the last part of the introduction, the author's summary of contributions is too brief and unclear. I suggest reorganizing this part to highlight the significance.

**Response 1：**

In the last part of the “Introduction”, we rewrote our contributions and highlighted the importance of the paper.

(2) In the results section, although the regression results confirm research hypothesis 1 and research hypothesis 2, I still recommend briefly summarizing research hypothesis 1 and research hypothesis 2 in this section. In addition, the authors also need to add appropriate economic meanings to explain what causes these results. This will make it easier for readers to understand.

**Response 2：**

In the “Results” section, we further specify the research hypothesis 1 and hypothesis 2 in combination with the regression results. We have also made corresponding economic explanations for the results so that the author can better understand them.

(3) The table of regression results is too large. I suggest leaving out unimportant control variables and constant items and replacing them with YES or NO.

**Response 3：**

In the regression tables, we remove the unimportant control variables and constant terms and replace them with YES or NO.

(4) Add contribution for body of knowledge in Conclusion section.

**Response 4：**

We added our contribution to the body of knowledge in the “Conclusion”.

(5) Add limitation and suggestion for future research in Conclusion section.

**Response 5：**

We added limitation and suggestion for future research in the “Conclusion”.
