# Supplementary material for: Private capital holding, financial awareness of government and steadying operation of banks: Evidence from China
Source: PLoS One. 2023 May 11;18(5):e0285588. doi: 10.1371/journal.pone.0285588 (PMC10174543; doi:10.1371/journal.pone.0285588)
Supplement: Attachment — Submitted filename: renamed_689f7.doc [file pone.0285588.s004.doc]

**审稿意见：大修**

问题1：在中国125家城市商业银行中，**广州银行、天津银行、北京银行、上海银行、齐鲁银行、南京银行、杭州银行、大连银行、盛京银行、长沙银行、重庆银行、成都银行、厦门银行等等**均是在省外异地设立分行的**城市商业银行**（比如，截至2011年9月，北京银行共设有上海、天津、杭州、深圳、西安、长沙、南京、济南、南昌9家分行，遍布中国9个省）。以文章中要加入GDP为例，按照作者的回复（因为城市商业银行在省内有多个分行，所以使用省级GDP，不能使用城市级GDP），那么上述银行必须使用跨省级GDP，甚至是中国GDP了，同理Finawa（政府的金融意识）也不能是省级指标了。

如果作者一定要加入宏观经济变量，审稿人提供几个解决方法，方法1是作者以城市商业银行总部所在的城市为参照物，采用城市级指标，这样的话必须从《中国城市年鉴》等相关数据库重新找数据，重新计算“GDP”、“Finawa”（政府的金融意识），并且文中要有相应的解释说明。方法2是删除这些跨省的城市商业银行，按照作者的想法，采用省级指标，但可能面临样本量变少，甚至样本量不够的情况。请作者自行斟酌选择。

当然，如果作者有更加合理的理由，认为数据不用重新收集或更改，审稿人认为“小修”就可以了，否则，必须“大修”

建议1：按照作者对审稿人2建议1的回复，政府的金融意识(Finawa)并不通过私人资本持有(Priv)影响银行(Ln_Z)的稳定运行，即不是中介效应，而是发挥的调节效应。在分析“Priv”对“Ln_Z”影响的基础上，进一步研究“Finawa”是否改变了“Priv”的影响，审稿人对此表示了认同。但作者的**研究假设2的标题“Financial awareness of government and steady operation of banks”**和研究假设2：**貌似没有体现“Finawa”的调节效应，而像是介绍****“Finawa”对steady operation of banks（ln-Z）的直接效应。**所以审稿人认为有必要对研究假设2的标题、研究假设2、甚至是内容分析做出修改。请作者进行修改。

建议2：按照审稿人2的建议3，作者在模型表达式中加入了“YEAR”，表示年份效应。但请注意措辞，Line 184 ：“Of course, we also control the trend effect of year, YEAR.”更改为“YEAR_t_表示年份效应”，Line 178：“W_i_ is the individual fixed effect of the banks”更改为“W_i_表示银行个体效应”，删掉“固定”两字。请作者进行修改。

建议3：按照审稿人2的建议6，作者增加了一小节“变量数据的平稳性检验”。但是按照作者的修改，Fisher检验指的是Fisher-ADF检验，还是Fisher-pp检验？请具体指明。并且Table3中，第一列应该列出所有变量的名称，并且应该前后文变量简写一致，“P”应更改为“Priv”、“Z”更改为“Ln-Z”，即要加对数，等等。审稿人甚至有种感觉，作者并没有真正进行变量平稳性检验，所以出现了这些错误。请作者进行修改。

建议4：审稿人2提的建议4，要对SYS-GMM方法的适用条件（如：随机扰动项自相关性检验、过度识别检验）进行说明，但审稿人发现作者未在文中阐述（虽然表格中有相关结果），请作者进行补充说明。审稿人认为这部分是有必要的，原因如下，文章的固定效应实证结果表明“Priv”的直接效应不显著，“Finawa”的调节效应不显著，而作者又想得出本文的研究结论，所以采用了SYS-GMM方法，在这里必须说明SYS-GMM方法的适用条件是否满足，这样本文研究的SYS-GMM的实证结果：“Priv”具有直接效应、“Finawa”具有调节效应才有理有据。请作者进行修改。

建议5：在“Table4-Table10”中，需增加“银行个体效应”；时间效应，应该为“年份效应”,而不是“year”；“control variables”应更改为“X”（因为“X”表示所有的控制变量），并且建议“X”、“年份效应”、“银行个体效应”在“Table 4—Table10”中分三行显示，实证结果用“Yes”表示。请作者进行修改。

建议6：请在本文的“Future prospects”中，指出本文研究的局限性，以此来引出该主题未来的研究方向。请作者补充说明。

Review comments: ***major revision***

**Question 1:** Among the 125 city commercial banks in China, the Bank of Guangzhou, the Bank of Tianjin, the Bank of Beijing, the Bank of Shanghai, the Bank of Qilu, the Bank of Nanjing, the Bank of Hangzhou, the Bank of Dalian, the Bank of Shengjing, the Bank of Changsha, the Bank of Chongqing, the Bank of Chengdu and the Bank of Xiamen, etc are all city commercial banks with branches outside the province. (For example, as of September 2011, Bank of Beijing has 9 branches in Shanghai, Tianjin, Hangzhou, Shenzhen, Xi 'an, Changsha, Nanjing, Jinan and Nanchang, covering 9 provinces in China.).Taking “GDP” as an example, according to the author's reply (since urban commercial banks have multiple branches in the province, provincial GDP is used instead of city-level GDP), the above banks must use cross-provincial GDP, even China's GDP. Similarly, “Finawa” (financial awareness of the government) cannot be a provincial index.

If the author must add macroeconomic variables, the reviewer provides several solutions. Method 1 is that the author takes the city where the headquarters of the city commercial bank is located as the reference and adopts city-level indicators. In this way, data must be found again from relevant databases such as China City Yearbook to recalculate "GDP" and "Finawa" (financial awareness of the government). And the text should have the corresponding explanation. Method 2 is to delete these inter-provincial city commercial banks and adopt provincial indicators according to the author's idea, but the sample size may be reduced or even insufficient. Please use your own discretion.

Of course, if the author has a more reasonable reason that the data need not be recollected or changed, the reviewer thinks that "minor revision" is OK, otherwise, it must be "major revision".

**Suggestion 1:** According to the author's reply to Suggestion 1 of Reviewer 2, the government's financial consciousness (Finawa) does not affect the stable operation of banks (Ln_Z) through private capital holding (Priv), that is, it is not an intermediary effect, but a moderating effect. On the basis of analyzing the influence of "Priv" on "Ln_Z", the reviewer further studies whether "Finawa" changes the influence of "Priv", which is agreed by the reviewer.

However, the title "Financial awareness of government and steady operation of banks" of the author's research hypothesis 2 and “research hypothesis 2” do not seem to reflect the moderating effect of "Finawa". Instead, introduce the direct effect of "Finawa" on the steady operation of banks (ln-Z). Therefore, the reviewer thinks it is necessary to revise the title of research hypothesis 2, research Hypothesis 2, and even the content analysis. Please modify it.

**Suggestion 2:** According to Suggestion 3 of Reviewer 2, the author added "YEAR" to “the model expression” to represent the year effect.

But please revise the wording. Line 184: "Of course, we also control the trend effect of year, YEAR." should be changed to "YEAR_t_ is year effect"; Line 178: "W_i_ is the individual fixed effect of the banks" is changed to "W_i_ is banks individual effect" and delete the word "fixed". Please modify it.

**Suggestion 3:** According to Suggestion 6 of Reviewer 2, the author adds a subsection "Stability test of Variable Data". But according to the author's modification, does “Fisher test” refer to “Fisher-ADF test” or “Fisher-PP test”? Please specify.

In Table 3, Column 1 should list the names of all variables, and the abbreviations of the variables before and after should be the same. For example, "P" should be changed to "Priv", "Z" should be changed to "Ln-Z", that is, to add logarithms, and so on. The reviewer even has the feeling that the author did not really test the Stability of the variables, which led to these errors. Please modify it.

**Suggestion 4:** According to Suggestion 4 of Reviewer 2, it is necessary to explain the application conditions of SYS-GMM method (such as autocorrelation test of random disturbance items and overrecognition test), but the reviewer found that the author did not explain in the paper (although there are relevant results in the table), please make a supplementary explanation.

The reviewer thinks this part is necessary for the following reasons: the empirical results of fixed effects of this paper show that the direct effect of "Priv" is not significant, and the moderating effect of "Finawa" is not significant, and the author wants to reach the research conclusion of this paper, so the SYS-GMM method is adopted. It must be explained here whether the application conditions of SYS-GMM method are met. In this way, the empirical results of SYS-GMM studied in this paper, “"Priv" has a direct effect and "Finawa" has a moderating effect”, are reasonable. Please modify it.

**Suggestion 5:** In "Table 4～Table 10", it is necessary to increase the "bank individual effect"; Time effect, should be "year effect", not "year"; The "control variables" should be changed to "X" (because "X" represents all control variables), and it is suggested that "X", "year effect" and "bank individual effect" should be displayed in three lines in "Table 4～Table 10", and the empirical results should be represented by "Yes". Please modify it.

**Suggestion 6:** In the "Future prospects" of this paper, please point out the limitations of this research, so as to lead to the future research direction of this topic. Please add clarification.
