# Supplementary material for: Private capital holding, financial awareness of government and steadying operation of banks: Evidence from China
Source: PLoS One. 2023 May 11;18(5):e0285588. doi: 10.1371/journal.pone.0285588 (PMC10174543; doi:10.1371/journal.pone.0285588)
Supplement: Attachment — Submitted filename: Response to reviewers.docx [file pone.0285588.s005.docx]

****Response to reviewer #2** comments**

Dear Reviewer Haisong Dong,

First, we thank you for your affirmation of our last revision. Secondly, your worries and suggestions this time are also crucial. According to your suggestions, we have made the following revisions on this manuscript:

Question 1: Among the 125 city commercial banks in China, the Bank of Guangzhou, the Bank of Tianjin, the Bank of Beijing, the Bank of Shanghai, the Bank of Qilu, the Bank of Nanjing, the Bank of Hangzhou, the Bank of Dalian, the Bank of Shengjing, the Bank of Changsha, the Bank of Chongqing, the Bank of Chengdu and the Bank of Xiamen, etc are all city commercial banks with branches outside the province. (For example, as of September 2011, Bank of Beijing has 9 branches in Shanghai, Tianjin, Hangzhou, Shenzhen, Xi 'an, Changsha, Nanjing, Jinan and Nanchang, covering 9 provinces in China.).Taking “GDP” as an example, according to the author's reply (since urban commercial banks have multiple branches in the province, provincial GDP is used instead of city-level GDP), the above banks must use cross-provincial GDP, even China's GDP. Similarly, “Finawa” (financial awareness of the government) cannot be a provincial index.
If the author must add macroeconomic variables, the reviewer provides several solutions. Method 1 is that the author takes the city where the headquarters of the city commercial bank is located as the reference and adopts city-level indicators. In this way, data must be found again from relevant databases such as China City Yearbook to recalculate "GDP" and "Finawa" (financial awareness of the government). And the text should have the corresponding explanation. Method 2 is to delete these inter-provincial city commercial banks and adopt provincial indicators according to the author's idea, but the sample size may be reduced or even insufficient. Please use your own discretion.
Of course, if the author has a more reasonable reason that the data need not be recollected or changed, the reviewer thinks that "minor revision" is OK, otherwise, it must be "major revision".

**Response 1：**

Trans-provincial operation is a fact, but it must be admitted that the bank's main business area is in the province where it is registered, as shown in the following table. The focus of bank operation is almost all in the province where it is registered. Only Bank of Shanghai and Bank of Tianjin account for a smaller proportion of local operations. Even the Bank of Beijing, Bank of Dalian and Bank of Ningbo, which have many trans-provincial institutions, have more than half of their local operations, almost 70%. Of course, this is the conclusion based on the 2020 data analysis. In the earlier years, the proportion of local operations was higher. Therefore, we believe that it is reasonable to build macro indicators based on provincial level. The use of provincial macroeconomic indicators is also a common method of existing research (Sun et al., 2013; Liu et al., 2022a; 2022b).

| Bank | The proportion of banks operating in local provinces in 2020 (calculated according to the regional distribution of total loans, total assets or operating income disclosed in the bank's annual report) |
| --- | --- |
| Bank of Beijing | 68% |
| Bank of Shanghai | 44.68% |
| Bank of Tianjin | 22.2% |
| Bank of Chongqing | 78.7% |
| Bank of Guangzhou | 96.1% |
| Qilu Bank | 94.04% |
| Bank of Nanjing | 82.43% |
| Bank of Hangzhou | 69.77% |
| Bank of Dalian | 55.44% |
| Shengjing Bank | 92.5% |
| Bank of Changsha | 97.16% |
| Bank of Chengdu | 93.33% |
| Xiamen Bank | 100% |
| Bank of Ningbo | 66.98% |
| Bank of Guiyang | 94.33% |
| Bank of Dongguan | 92.02% |
| Huishang Bank | 78.15% |
| Bank of Jiangsu | 82.54% |

We removed the control variable (*GDP*) in the regression in order to prove that the selection of macroeconomic indicators is reasonable. The regression results are shown in the following table. It can be found that the regression results are still robust and reliable.

|  | (1) | (2) | (3) | (4) | (5) | (6) | (7) | (8) |
| --- | --- | --- | --- | --- | --- | --- | --- | --- |
|  | *Ln_Z* | *Ln_Z* | *ROA* | *ROA* | *CAR* | *CAR* | *σ(ROA)* | *σ(ROA)* |
| *Priv* | 0.508^***^ | 1.577^***^ | 0.211^***^ | 0.538^***^ | -0.037^***^ | -0.184^***^ | -0.153^**^ | -0.222^***^ |
|  | (3.46) | (6.01) | (10.32) | (2.84) | (-3.82) | (-7.81) | (-2.54) | (-3.79) |
| *Finawa* |  | -0.007 |  | 0.225^***^ |  | -0.001 |  | -0.148^***^ |
|  |  | (-0.03) |  | (2.99) |  | (-0.17) |  | (-4.44) |
| *Priv×Finawa* |  | -1.700^***^ |  | -0.550^**^ |  | 0.227^***^ |  | 0.168^*^ |
|  |  | (-4.03) |  | (-2.29) |  | (7.19) |  | (1.93) |
| *Ln_Z_t-1_* | 0.879^***^ | 0.808^***^ |  |  |  |  |  |  |
|  | (18.18) | (20.13) |  |  |  |  |  |  |
| *ROA_t-1_* |  |  | 0.342^***^ | 0.405^***^ |  |  |  |  |
|  |  |  | (13.13) | (17.63) |  |  |  |  |
| *CAR_t-1_* |  |  |  |  | 0.204^***^ | 0.173^***^ |  |  |
|  |  |  |  |  | (10.44) | (22.19) |  |  |
| *σ(ROA)_t-1_* |  |  |  |  |  |  | 0.659^***^ | 0.402^***^ |
|  |  |  |  |  |  |  | (11.56) | (24.58) |
| bank individual effect/year effect/*X* | Yes | Yes | Yes | Yes | Yes | Yes | Yes | Yes |
| *N* | 466 | 466 | 658 | 658 | 681 | 681 | 480 | 480 |
| Wald | 582.42 | 2728.76 | 1327.14 | 1465.76 | 215.35 | 912.05 | 352.83 | 1721.09 |
|  | 0.0000 | 0.0000 | 0.0000 | 0.0000 | 0.0000 | 0.0000 | 0.0000 | 0.0000 |
| Ar(1)-p | 0.0000 | 0.0000 | 0.0062 | 0.0076 | 0.0531 | 0.0492 | 0.0016 | 0.0002 |
| Ar(2)-p | 0.4615 | 0.4519 | 0.3576 | 0.1849 | 0.3738 | 0.5107 | 0.0513 | 0.3706 |
| Sargan-p | 0.7268 | 0.2196 | 0.3339 | 0.3856 | 0.4661 | 0.1156 | 0.3812 | 0.5513 |

Further, we removed the sample of CCBs (Bank of Shanghai, Bank of Tianjin, Bank of Beijing, Bank of Dalian and Bank of Ningbo) with a large proportion of operations in other provinces. The regression results are shown in the following table. It can be found that the regression results are still robust and reliable.

|  | (1) | (2) | (3) | (4) | (5) | (6) | (7) | (8) |
| --- | --- | --- | --- | --- | --- | --- | --- | --- |
|  | *Ln_Z* | *Ln_Z* | *ROA* | *ROA* | *CAR* | *CAR* | *σ(ROA)* | *σ(ROA)* |
| *Priv* | 0.505^***^ | 1.449^***^ | 0.188^***^ | 0.523^***^ | -0.027^***^ | -0.184^***^ | -0.149^**^ | -0.244^***^ |
|  | (3.37) | (6.33) | (6.82) | (2.90) | (-2.71) | (-8.30) | (-2.45) | (-5.45) |
| *Finawa* |  | 0.115 |  | 0.257^***^ |  | -0.007 |  | -0.018 |
|  |  | (0.66) |  | (3.40) |  | (-1.59) |  | (-0.45) |
| *Priv×Finawa* |  | -1.560^***^ |  | -0.521^**^ |  | 0.226^***^ |  | 0.160^*^ |
|  |  | (-4.20) |  | (-2.26) |  | (7.72) |  | (1.88) |
| *Ln_Z_t-1_* | 0.889^***^ | 0.793^***^ |  |  |  |  |  |  |
|  | (18.62) | (24.73) |  |  |  |  |  |  |
| *ROA_t-1_* |  |  | 0.354^***^ | 0.391^***^ |  |  |  |  |
|  |  |  | (12.70) | (17.53) |  |  |  |  |
| *CAR_t-1_* |  |  |  |  | 0.199^***^ | 0.172^***^ |  |  |
|  |  |  |  |  | (9.73) | (22.26) |  |  |
| *σ(ROA)_t-1_* |  |  |  |  |  |  | 0.649^***^ | 0.581^***^ |
|  |  |  |  |  |  |  | (11.57) | (64.10) |
| bank individual effect/year effect/*X* | Yes | Yes | Yes | Yes | Yes | Yes | Yes | Yes |
| *N* | 442 | 442 | 625 | 625 | 648 | 648 | 456 | 456 |
| Wald | 692.08 | 3735.11 | 1139.48 | 1535.15 | 199.80 | 953.03 | 363.88 | 14594.75 |
|  | 0.0000 | 0.0000 | 0.0000 | 0.0000 | 0.0000 | 0.0000 | 0.0000 | 0.0000 |
| Ar(1)-p | 0.0000 | 0.0000 | 0.0107 | 0.0085 | 0.0584 | 0.0488 | 0.0013 | 0.0004 |
| Ar(2)-p | 0.2148 | 0.2207 | 0.5133 | 0.2053 | 0.4779 | 0.5442 | 0.0537 | 0.2330 |
| Sargan-p | 0.8019 | 0.2558 | 0.4251 | 0.4626 | 0.3159 | 0.1653 | 0.2633 | 0.5322 |

Suggestion 1: According to the author's reply to Suggestion 1 of Reviewer 2, the government's financial consciousness (Finawa) does not affect the stable operation of banks (Ln_Z) through private capital holding (Priv), that is, it is not an intermediary effect, but a moderating effect. On the basis of analyzing the influence of "Priv" on "Ln_Z", the reviewer further studies whether "Finawa" changes the influence of "Priv", which is agreed by the reviewer.
However, the title "Financial awareness of government and steady operation of banks" of the author's research hypothesis 2 and “research hypothesis 2” do not seem to reflect the moderating effect of "Finawa". Instead, introduce the direct effect of "Finawa" on the steady operation of banks (ln-Z). Therefore, the reviewer thinks it is necessary to revise the title of research hypothesis 2, research Hypothesis 2, and even the content analysis. Please modify it.

**Response 1：**

We have revised the title of research hypothesis 2 and research Hypothesis 2.

Suggestion 2: According to Suggestion 3 of Reviewer 2, the author added "YEAR" to “the model expression” to represent the year effect.
But please revise the wording. Line 184: "Of course, we also control the trend effect of year, YEAR." should be changed to "YEARt is year effect"; Line 178: "Wi is the individual fixed effect of the banks" is changed to "Wi is banks individual effect" and delete the word "fixed". Please modify it.

**Response 2：**

We have revised the relevant words.

Suggestion 3: According to Suggestion 6 of Reviewer 2, the author adds a subsection "Stability test of Variable Data". But according to the author's modification, does “Fisher test” refer to “Fisher-ADF test” or “Fisher-PP test”? Please specify.
In Table 3, Column 1 should list the names of all variables, and the abbreviations of the variables before and after should be the same. For example, "P" should be changed to "Priv", "Z" should be changed to "Ln-Z", that is, to add logarithms, and so on. The reviewer even has the feeling that the author did not really test the Stability of the variables, which led to these errors. Please modify it.

**Response 3：**

Our "Fisher test" refers to "Fisher - ADF test". We did not specify the meaning of the symbols in the first column of Table 3 in detail in the manuscript, which is easy to be misunderstood. Here, P, Z, L and Pm are the four statistics of "Fisher - ADF test", namely Inverse Chi-squared P, Inverse normal Z, Inverse logit t L and Modified inverse chi-squared Pm, not variables. We have added relevant instructions to the manuscript.

Suggestion 4: According to Suggestion 4 of Reviewer 2, it is necessary to explain the application conditions of SYS-GMM method (such as autocorrelation test of random disturbance items and overrecognition test), but the reviewer found that the author did not explain in the paper (although there are relevant results in the table), please make a supplementary explanation.
The reviewer thinks this part is necessary for the following reasons: the empirical results of fixed effects of this paper show that the direct effect of "Priv" is not significant, and the moderating effect of "Finawa" is not significant, and the author wants to reach the research conclusion of this paper, so the SYS-GMM method is adopted. It must be explained here whether the application conditions of SYS-GMM method are met. In this way, the empirical results of SYS-GMM studied in this paper, “"Priv" has a direct effect and "Finawa" has a moderating effect”, are reasonable. Please modify it.

**Response 4：**

We have explained the application conditions of the SYS-GMM method based on the regression results in Table 4 and Table 5.

Suggestion 5: In "Table 4～Table 10", it is necessary to increase the "bank individual effect"; Time effect, should be "year effect", not "year"; The "control variables" should be changed to "X" (because "X" represents all control variables), and it is suggested that "X", "year effect" and "bank individual effect" should be displayed in three lines in "Table 4～Table 10", and the empirical results should be represented by "Yes". Please modify it.

**Response 5：**

We have revised the presentation of regression results.

Suggestion 6: In the "Future prospects" of this paper, please point out the limitations of this research, so as to lead to the future research direction of this topic. Please add clarification.

**Response 6：**

We have added the limitations of the paper.

Sun J, Harimaya K, Yamori N. Regional economic development, strategic investors, and efficiency of Chinese city commercial banks. Journal of Banking & Finance. 2013; 37(5): 1602-1611. https://doi.org/10.1016/j.jbankfin.2012.12.013

Liu J, Xu L, Zhang Q. The influence of the largest private shareholder on bank loans: Evidence from China. PLoS ONE. 2022a; 17(10): e0276877. https://doi.org/10.1371/journal.pone.0276877

Liu J, Zhang Q, Xiao C. Large private shareholders, industrial policies and industrial loans of city commercial banks: Evidence from China. PLoS ONE. 2022b; 17(12): e0278654. https://doi.org/10.1371/journal.pone.0278654

****Response to reviewer #3** comments**

Dear Qihui Chen,

We are encouraged by your approval of our revision. We express our profound gratitude for this. The problem of sample self-selection is the key to the reliability of empirical analysis, and we attach great importance to it. Our response to this problem is as follows:

In addition to the influence of the lag-term set by the model, the other reason is that the Z value is obtained from the moving average of the three years before and after the *ROA* and *CAR*, which results in the Z value of the first and last year of the sample being the missing value, so the observations of *Ln_Z* is less than that of *ROA* and *CAR*. The same is true of Volatility of *ROA*. There is no serious sample self-selection problem in this processing method.

However, in order to increase the reliability of the empirical results, we test whether there is a problem of sample self-selection according to the idea of Beckman (1979) two-stage estimation. The results are shown in the following table.

The regression results of the first stage are consistent with the first column of Table 8 in the manuscript. By calculating the Inverse Mill Ratio index (*IMR*) and adding it to the second stage of regression, the results show that the coefficients of *IMR* are not significant, indicating that there is no serious problem of sample self-selection.

|  | (1) | (2) | (3) | (4) | (5) |
| --- | --- | --- | --- | --- | --- |
|  | *Priv* | *Ln_Z* | *ROA* | *CAR* | *σ(ROA)* |
| *Priv* |  | 0.490^***^ | 0.232^***^ | -0.029^***^ | -0.139^**^ |
|  |  | (3.33) | (7.99) | (-3.48) | (-2.27) |
| *Ln_Z_t-1_* |  | 0.875^***^ |  |  |  |
|  |  | (19.16) |  |  |  |
| *ROA_t-1_* |  |  | 0.380^***^ |  |  |
|  |  |  | (14.27) |  |  |
| *CAR_t-1_* |  |  |  | 0.175^***^ |  |
|  |  |  |  | (14.75) |  |
| *σ(ROA)_t-1_* |  |  |  |  | 0.675^***^ |
|  |  |  |  |  | (14.69) |
| *Priv_iv* | 0.499^**^ |  |  |  |  |
|  | (2.22) |  |  |  |  |
| *Finawa* | 0.634^***^ |  |  |  |  |
|  | (5.87) |  |  |  |  |
| *IMR* |  | -1.271 | 0.134 | -0.014 | 0.435 |
|  |  | (-1.34) | (0.63) | (-0.48) | (1.47) |
| bank individual effect/year effect/*X* | Yes | Yes | Yes | Yes | Yes |
| *N* | 694 | 466 | 657 | 680 | 480 |
| Wald |  | 738.29 | 1187.63 | 393.41 | 606.25 |
|  |  | 0.0000 | 0.0000 | 0.0000 | 0.0000 |
| Ar(1)-p |  | 0.0000 | 0.0073 | 0.0619 | 0.0009 |
| Ar(2)-p |  | 0.4269 | 0.4861 | 0.6445 | 0.3460 |
| Sargan-p |  | 0.5345 | 0.4014 | 0.1718 | 0.0667 |

Heckman J. Sample selection bias as a specification error. Econometrica. 1979; 47(1): 153-161. http://dx.doi.org/10.2307/1912352
