# Supplementary material for: Private capital holding, financial awareness of government and steadying operation of banks: Evidence from China
Source: PLoS One. 2023 May 11;18(5):e0285588. doi: 10.1371/journal.pone.0285588 (PMC10174543; doi:10.1371/journal.pone.0285588)
Supplement: Attachment — Submitted filename: renamed_eb103.doc [file pone.0285588.s006.doc]

**审稿意见：小修**

谢谢作者对审稿人问题和建议的说明和修改，但有些细节或部分尚待改进，具体如下：

建议1：研究假设2的标题建议改成“Financial awareness of government，Private capital holding，and steady operation of banks”，一方面可以体现出“Finawa”的调节效应，另一方面与研究假设1的标题“Ownership structure and steady operation of banks”格式一致。

或者保留现有的研究假设2的标题“The moderating effect of the government’s financial awareness”不变，研究假设1的标题改为“Private capital holding的直接影响”，这样可能格式更一致。请作者斟酌考虑。

建议2：第183行：“Of course, we also control the year effect, *Y EAR*.”，作者用YEAR控制时间效应，这种表达，YEAR应该是时间趋势项，模型表达式中便不是“YEAR_t_”，而应该是“λYEAR”，即“YEAR”前应有常数项λ；相应的“Table 4—Table10”，“year effect”应改为“YEAR”.如果第183行更改为“YEAR_t_ is year effect”，表示“YEAR_t_”就是时间效应，模型表达式不变，相应的“Table 4—Table10”，“year effect”也不变。作者结合自身实证结果，确认是“YEAR”还是“YEAR_t_”，做出对应的修改。

建议3：“Stability test of variable”，应该是所有变量都要进行平稳性检验，即所有变量都要进行Fisher-ADF检验，所有变量都平稳或所有变量间存在协整关系，才可以避免“伪回归”，保证实证分析的准确性。而不应该只进行“*Ln Z*”的Fisher-ADF检验，请作者修改。

建议4：SYS-GMM方法的适用条件之一：随机扰动项的自相关检验是通过随机扰动项的差分是否存在一阶与二阶自相关来检验的，只有随机扰动项的差分存在一阶自相关但不存在二阶自相关，才满足SYS-GMM方法的适用条件，所以请作者在第229行中补充说明随机扰动项的差分存在一阶自相关。请作者补充阐述。

另外，第231行：This means that the random disturbance term has no autocorrelation and pass the over-identification test.翻译有歧义，请作者修改。

建议5：“Results”小节，Table 4应该去掉，相关Table 4的分析也可以去掉，原因在于，一、Table 5可以作为本文的基准回归结果，既可以进行研究假设2的验证，也可以进行研究假设1的验证。Table 4就多余了。二、“Robustness checks”小节进行的两个稳定性检验，也是将Table 5作为基准回归结果进行比较检验的，这样也说明Table 4多余了。另外需要在Table 5中补充研究假设1的验证分析。请作者修改。

Review comments: ***minor revision***

Thank the authors for explaining and modifying of the reviewer's questions and suggestions, but some details or parts need to be improved, specifically as follows:

**Suggestion 1:** The title of hypothesis 2 is suggested to be revised to "Financial awareness of government, Private capital holding, and steady operation of banks". On the one hand, it can reflect the moderating effect of "Finawa"; on the other hand, it is consistent with the format of the title "Ownership structure and steady operation of banks" in hypothesis 1.

Or the title of hypothesis 2, "The moderating effect of the government's financial awareness", should be retained unchanged. The title of research hypothesis 1 is modified to "Direct Impact of Private capital holding", which may be more consistent in format. Please consider.

**Suggestion 2:** Line 183:"Of course, we also control the year effect, YEAR.", the author uses “YEAR” to control the time effect, this expression, “YEAR” should be the time trend term, the model expression is not "YEAR_t_", but "λYEAR", that is, there should be a constant term λ before "YEAR"; Corresponding "Table 4-Table10", "year effect" should be modified to "YEAR".

If line 183 is modified to "YEAR_t_ is year effect", it means that "YEAR_t_" is the time effect, the model expression is unchanged, and the corresponding "Table 4-Table10", "year effect" is also unchanged. Based on the empirical results, the authors confirm whether it is "YEAR" or "YEAR_t_" and make corresponding modifications. Please modify it.

**Suggestion 3:** In the "Stability test of variable" section, it should be that all variables should undergo the stability test, that is, all variables should undergo the Fisher-ADF test.

Only when all variables are stationary or there is a co-integration relationship between all variables can "pseudo-regression" be avoided and the accuracy of empirical analysis be guaranteed. Authors should not just perform Fisher-ADF tests for "Ln Z". Please modify it.

**Suggestion 4:** One of the applicable conditions of SYS-GMM method is the autocorrelation test of random disturbance terms, which is tested by whether the difference of random disturbance terms exists first-order and second-order autocorrelation. The autocorrelation test of random disturbance item can only be passed if the difference of random disturbance item has first-order autocorrelation but not second-order autocorrelation. Therefore, the authors should add that the difference of random disturbance item has first-order autocorrelation in Line 229. Please add clarification.

Also, “This means that the random disturbance term has no autocorrelation and pass the over-identification test.”（Line 231）is some ambiguity in the translation. Please modify it.

**Suggestion 5:** In section "Results", Table 4 should be removed, as well as the analysis related to Table 4. The reason is that, firstly, Table 5 can be used as the benchmark regression result of this paper, which can be used to verify both hypothesis 2 and hypothesis 1. So table 4 is redundant. Secondly, the two stability tests carried out in the subsection "Robustness checks" are also comparing with Table 5 as the benchmark regression results, which also mean that Table 4 is redundant. In addition, validation analysis of research hypothesis 1 should be supplemented in Table 5. Please modify it.
