# Supplementary material for: Private capital holding, financial awareness of government and steadying operation of banks: Evidence from China
Source: PLoS One. 2023 May 11;18(5):e0285588. doi: 10.1371/journal.pone.0285588 (PMC10174543; doi:10.1371/journal.pone.0285588)
Supplement: Attachment — Submitted filename: Response to reviewers.docx [file pone.0285588.s007.docx]

****Response to reviewer #2** comments**

Dear Reviewer Haisong Dong,

Once again, thank you for your affirmation of the revisions we have made. Your suggestions are really helpful for revising and improving our paper. According to your suggestions, we have made the following revisions on this manuscript:

Suggestion 1: The title of hypothesis 2 is suggested to be revised to "Financial awareness of government, Private capital holding, and steady operation of banks". On the one hand, it can reflect the moderating effect of "Finawa"; on the other hand, it is consistent with the format of the title "Ownership structure and steady operation of banks" in hypothesis 1.
Or the title of hypothesis 2, "The moderating effect of the government's financial awareness", should be retained unchanged. The title of research hypothesis 1 is modified to "Direct Impact of Private capital holding", which may be more consistent in format. Please consider.

**Response 1：**

We have changed the title of hypothesis 2 to "Financial awareness of government, Private capital holding, and steady operation of banks".

Suggestion 2: Line 183:"Of course, we also control the year effect, YEAR.", the author uses “YEAR” to control the time effect, this expression, “YEAR” should be the time trend term, the model expression is not "YEARt", but "λYEAR", that is, there should be a constant term λ before "YEAR"; Corresponding "Table 4-Table10", "year effect" should be modified to "YEAR".
If line 183 is modified to "YEARt is year effect", it means that "YEARt" is the time effect, the model expression is unchanged, and the corresponding "Table 4-Table10", "year effect" is also unchanged. Based on the empirical results, the authors confirm whether it is "YEAR" or "YEARt" and make corresponding modifications. Please modify it.

**Response 2：**

The *YEAR* here is the year effect, which we labeled "*year_t_*".

Suggestion 3: In the "Stability test of variable" section, it should be that all variables should undergo the stability test, that is, all variables should undergo the Fisher-ADF test.
Only when all variables are stationary or there is a co-integration relationship between all variables can "pseudo-regression" be avoided and the accuracy of empirical analysis be guaranteed. Authors should not just perform Fisher-ADF tests for "Ln Z". Please modify it.
**Response 3：**

We have added stability test for other variables.

Suggestion 4: One of the applicable conditions of SYS-GMM method is the autocorrelation test of random disturbance terms, which is tested by whether the difference of random disturbance terms exists first-order and second-order autocorrelation. The autocorrelation test of random disturbance item can only be passed if the difference of random disturbance item has first-order autocorrelation but not second-order autocorrelation. Therefore, the authors should add that the difference of random disturbance item has first-order autocorrelation in Line 229. Please add clarification.
Also, “This means that the random disturbance term has no autocorrelation and pass the over-identification test.”（Line 231）is some ambiguity in the translation. Please modify it.

**Response 4：**

As you pointed out, the prerequisite of SYS-GMM is that there is no autocorrelation in the differences of random disturbance terms. Of course, the most critical test is to verify whether there is second-order autocorrelation. Theoretically, even if the assumption is satisfied, there still is a first-order autocorrelation, but in fact, due to the factual situation of the data, the first-order autocorrelation may not exist.

Meanwhile, we replaced “This means that the random disturbance term has no autocorrelation and pass the over-identification test.” with “This means that the random disturbance term has no autocorrelation, and the model setting passes the over-identification test.”

Suggestion 5: In section "Results", Table 4 should be removed, as well as the analysis related to Table 4. The reason is that, firstly, Table 5 can be used as the benchmark regression result of this paper, which can be used to verify both hypothesis 2 and hypothesis 1. So table 4 is redundant. Secondly, the two stability tests carried out in the subsection "Robustness checks" are also comparing with Table 5 as the benchmark regression results, which also mean that Table 4 is redundant. In addition, validation analysis of research hypothesis 1 should be supplemented in Table 5. Please modify it.

**Response 5：**

Indeed, Table 5 is more important than Table 4, but we need Table 4 to directly confirm Hypothesis 1. In contrast, the role of Table 5 is to reflect regulatory effects. Therefore, we would like you to agree that we retain Table 4.
