# Supplementary material for: Private capital holding, financial awareness of government and steadying operation of banks: Evidence from China
Source: PLoS One. 2023 May 11;18(5):e0285588. doi: 10.1371/journal.pone.0285588 (PMC10174543; doi:10.1371/journal.pone.0285588)
Supplement: Attachment — Submitted filename: Response to reviewers.docx [file pone.0285588.s008.docx]

****Response to reviewer #2** comments**

Dear Reviewer Haisong Dong,

Thank you again for your suggestion. Your suggestions are really helpful for revising and improving our paper. According to your suggestions, we have made the following revisions on this manuscript:

Suggestion 1: In Line 184, please change "Of course, we also control the year effect, yeart." to "Of course, yeart is year effect."

**Response 1：**

We have made this change.

Suggestion 2: In Line 232, please change “Arellano-Bond test for zero autocorrelation in first-differenced errors shows that all Ar(2)-p are greater than 0.05,” to “In the Arellano-Bond test, the difference of random disturbance terms shows that all Ar(1)-p is less than 0.1, and all Ar(2)-p is greater than 0.1, that is, the difference of random disturbance terms has first-order autocorrelation and no second-order autocorrelation;”

**Response 2：**

We have made this change.

Suggestion 3: In the section "Results", Table 4 should be removed, as well as related analysis in Table 4. In addition, verification analysis of research hypothesis 1 should be supplemented in Table 5. The reasons are as follows: First, Table 5 can be used as the benchmark regression result of this paper, which can be used for the verification of Hypothesis 2 and Hypothesis 1. Second, The two stability tests carried out in the section "Robustness checks" are also carried out with Table 5 as the benchmark regression results for comparative testing.
**Response 3：**

We have made this change. The specific content is reflected in lines 206 to 246 of the revised manuscript.
